# Regulation of the Electroanalytical Performance of Ultrathin Titanium Dioxide Nanosheets toward Lead Ions by Non-Metal Doping

**DOI:** 10.3390/nano7100327

**Published:** 2017-10-14

**Authors:** Junping Zhang, Jianjun Liao, Fan Yang, Ming Xu, Shiwei Lin

**Affiliations:** 1 State Key Laboratory of Marine Resource Utilization in South China Sea, Hainan University, Haikou 570228, China; ping18389597066@163.com (J.Z.); liaojianjun008@163.com (J.L.); 18789276621@163.com (F.Y.); xumingsunny1995@163.com (M.X.); 2 College of Materials and Chemical Engineering, Hainan University, Haikou 570228, China; 3 Institute of Tropical Agriculture and Forestry, Hainan University, Haikou 570228, China

**Keywords:** TiO_2_ nanosheets, fluorine doping, electrochemical detection, heavy metal ions, theoretical calculations

## Abstract

Three non-metallic elements, sulfur, fluorine, and iodine, were used to dope the ultrathin two-dimensional TiO_2_ nanosheets, which would regulate their electroanalytical properties toward heavy metal ions. Among these doped materials, fluorine-doped TiO_2_ nanosheets shows the highest electrochemical sensitivity and a superior detection limit toward Pb(II) when the doping concentration is 10%. When compared with the bare TiO_2_ nanosheets, the sensitivity increased by 102%, and the detection limit decreased by 36.4%. Through combining further electrochemical experiments and density-functional theory calculations, the enhanced electrochemical performance stemming from element doping was then investigated in detail. The theoretical calculation demonstrated that fluorine doping could greatly increase the adsorption energy of Pb(II) on the TiO_2_ nanosheets and enhance their loading capacity. Both cyclic voltammetric and electrical impedance spectroscopy analysis indicated the enhanced electron transfer rate on the electrode modified by fluorine-doped TiO_2_ nanosheets. Further measurement on the desorption performance showed the better stripping response of Pb(II) on the electrode with TiO_2_ nanosheets after fluorine doping, which suggests that fluorine doping is beneficial for Pb(II) diffuse onto the electrode surface for the reduction and stripping reaction. Therefore, the element doping of two-dimensional TiO_2_ nanosheets provides a facile method to extend the electronic materials toward detection of heavy metal ions in the environment.

## 1. Introduction

Heavy metal ions (HMIs), such as lead, mercury, cadmium, copper, zinc, and cadmium, etc., can accumulate in the ecosystems, which bring about adverse effects not only in the environment but also on human health by posing a serious damage to the immune, central nervous and reproductive systems even if the presence of trace amounts of them [1,2]. Therefore, there is an urgent need to search for a highly sensitivity, simple, and rapid detection means. In recent years, quite a few of analytical methods have been developed to detect HMIs, such as atomic absorption spectroscopy (AAS) [3], inductively coupled plasma mass spectrometry (ICP-MS) [4], atomic fluorescence spectrometry (AFS) [5], and inductively coupled plasma atomic emission spectrometry (ICP-AES) [6], and circular dichroism (CD) spectrometry [7,8], etc. However, most of these methods require cumbersome pre-processing and complicated instrumentation, so that they are not suitable for real-time on-site analysis. In contrast, the electrochemical method, especially anodic stripping voltammetry (ASV) has evolved into a very effective means toward HMIs detection due to its excellent sensitivity, well-defined peaks, easy operation, fast response, low cost, and portability. More importantly, it can also detect several HMIs simultaneously.

For the ASV methods, the enhancement of sensitivity and selectivity can be achieved by proper choice of the working electrode or rational design of the electrode surface [9,10,11,12]. Early mercury-based electrodes were frequently carried out due to the unique ability of mercury to preconcentrate heavy metals [9]. However, when considering its serious toxicity and difficulty in handling, great efforts have been devoted to search for excellent sensing materials to replace this electrode, such as carbon-based materials [13,14,15], mesoporous silica [16], metal oxides (F_3_O_4_, Cu_2_O, Co_3_O_4_, etc.) [1,17,18], and novel metal nanoparticle modifications [2,19,20,21]. Recently, graphene-like two-dimensional TiO_2_ nanosheets have received great concern ascribed to their large specific surface area, clear surface atom arrangement, confined atomic level thickness, and rich reaction activity [22,23,24]. Their performances in energy conversion and storage applications have been widely studied, including water splitting catalysis [23], and sodium-ion batteries [24,25], etc. However, few studies on the electroanalytical properties of ultrathin TiO_2_ nanosheets were reported. Besides, element doping is an effective approach to adjust the physicochemical properties of nanomaterials [26]. For example, non-metal (N, S, P, etc.) doped TiO_2_ could regulate the band-gap to enhance light absorption and photocatalytic activity [27,28,29]. Initiated by this view, by introducing a foreign element into the lattice of TiO_2_, the electronic structure would be regulated, which might be favorable to achieve specific electrochemical analysis properties [30]. 

In this work, non-metal doped ultrathin TiO_2_ nanosheets (TiO_2_ NSs) were synthesized by one-step hydrothermal method, and sulfur, fluorine, and iodine were individually introduced as the doping element by adding the corresponding source into the precursor. The morphology and structure of TiO_2_ nanosheets were characterized by transmission electron microscopy (TEM), X-ray diffraction (XRD), and X-ray photoelectron spectra (XPS). Anodic stripping voltammetry (ASV) was applied to evaluate the electrochemical performance of the electrodes modified with various TiO_2_ nanosheets toward lead ion detection. In order to clarify the contribution mechanism of element doping, the cyclic voltammograms (CV), electrochemical impedance performance (EIS), and stripping response were further investigated. The first-principles calculations were also performed to study the principle and mechanism of the adsorption behavior of Pb(II) on TiO_2_ nanosheets with/without fluorine doping in atomic level system, which could provide a deep understanding of the modification effect of the element doping on the electrochemical performance.

## 2. Experimental Section

### 2.1. Chemicals

All of the chemicals used in this study were of analytical grade and were used as received without any further purification from Sinopharm Chemical Reagent Co., Ltd., Shanghai, China. Stock solution of Pb(II) was prepared by dissolving the appropriate amounts of Pb(NO_3_)_2_ in deionized water. 0.1 M Acetate buffer solutions (NaAc-HAc) with different pH values were prepared by mixing stock solutions of 0.1 M NaAc and HAc. Phosphate buffer solution (PBS) of 0.1 M was prepared by mixing stock solutions of 0.1 M H_3_PO_4_, KH_2_PO_4_, K_2_HPO_4_, and NaOH. Ammonium chloride buffer solution (NH_4_Cl-HCl) of 0.1 M was prepared by mixing stock solutions of 0.1 M hydrochloric acid and ammonia. Ultrapure fresh water was obtained from the water purification system (specific resistivity = 18.25 MΩ·cm) and used in all runs. 

### 2.2. Preparation of Pure and Non-Metal Doped Ultrathin 2D TiO_2_ Nanosheets

The ultrathin two-dimensional (2D) TiO_2_ nanosheets (TiO_2_ NSs) were prepared according to a modified hydrothermal method [22]. Briefly, 2.10 g titanium isopropoxide (TTIP) and 1.48 g concentrated HCl solution was mixed together (bottle A); and 0.4 g pluronic (P123) was added into 6.0 g ethanol (bottle B). After separately stirring for 15 min, bottle A and bottle B was mixed and stirred for additional 30 min. Then, 5.0 mL the resulting solution was introduced into 40 mL ethylene glycol and transferred into a 100 mL dried Teflon-lined autoclave, which was maintained at 150 °C for 20 h. Afterward, the obtained solution was centrifuged at 10,000 rpm for 5 min and washed with ethanol and distilled water several times to remove the residual organic solvent. Finally, the obtained powder was collected as the pure TiO_2_ nanosheets after vacuum drying at 80 °C for 24 h. Meanwhile, the experimental procedures for fluorine-doped TiO_2_ were similar to the preparation of TiO_2_ except that for a certain proportion of sodium, fluoride was added into bottle B as fluorine doping precursor with the molar ratio of NaF to TTIP as 0.05, 0.1, and 0.15 (the obtained materials were referred to as 5% F-TiO_2_, 10% F-TiO_2_, 15% F-TiO_2_ nanosheets). For sulfur and iodine doped TiO_2_ nanosheets (denoted as S-TiO_2_ and I-TiO_2_, respectively), the preparation conditions were the same except that sodium iodide and thiourea were used as the doping precursors, respectively.

### 2.3. Electrode Fabrication

Before modification, the bare glass carbon electrode (GCE) was sequentially polished with 0.3 μm and 0.05 μm alumina power slurries to a mirror shiny surface, and then successively sonicated with HNO_3_ solution, ethanol, deionized water sequentially for 3 min to eliminate the effect of interfering substances on the electrode surface. As-prepared 3 mg ultrathin 2D TiO_2_ nanosheets were dissolved into 1 mL ethanol to obtain a homogeneous suspension. Afterwards, 3 μL of the suspension was pipetted onto the GCE surface. TiO_2_ NSs modified GCE was then obtained when the solvent evaporated at ambient temperature. Similarly, different concentrations of element doped TiO_2_ modified electrodes were prepared.

### 2.4. Materials Characterization Techniques

X-ray diffraction (XRD, Bruker D8 ADVANCE, Karlsruhe, Germany) analysis was recorded on diffractometer with Cu-Kα radiation. The surface morphology was obtained with TEM (JEM-2100, JEOL Ltd., Tokyo, Japan). X-ray photoelectron spectroscopy (XPS) was performed using a Thermo Escalab 250Xi (Thermo Fisher Scientific, Waltham, MA, USA) system using monochromatic Al Kα source. Specific surface area and pore size distribution were measured by a surface area analyzer (JW-BK112, JWGB Sci ＆ Tech Co.,Ltd., Beijing, China).

### 2.5. Electrochemical Experiments

All of the electrochemical tests were recorded on a CHI660D electrochemical workstation (ChenHua Instruments, Shanghai, China) and performed in a conventional three-electrode system composed of a bare or modified GCE (d = 3 mm) as the working electrode, Pt wire as the counter electrode, and an Ag/AgCl (3.5 M KCl) electrode as the reference electrode. CV and EIS analysis were recorded in 5 mM [Fe(CN)_6_]^3−/4−^ solution including 0.1 M KCl.

Square wave anodic stripping voltammetry (SWASV) was investigated under optimal experimental conditions. Lead ions were deposited at the potential of −1.0 V for 150 s in 0.1 M NaAc-HAc (pH 5.0). The anodic stripping of electrodeposited metal was measured at the following optimized conditions: step potential, 4 mV; amplitude, 25 mV; frequency, 15 Hz. Before testing, the electrodes were regenerated at 300 mV for 150 s to eliminate the residual metal ions from the previous measurement under stirring conditions.

### 2.6. Computational Details

First-principles calculations in this work were implemented using the Vienna ab-initio simulation package (VASP) program (Materials Design Inc., Vienna, Austria) that is based on density functional theory (DFT), which employs projector-augmented wave (PAW) pseudopotential to calculate the total energy [31,32,33,34]. We used the generalized gradient approximation (GGA) with the Perdew-Burke-Ernzerhof formulation (PBE) to treat the exchange and correlation energies [35]. The ion-electron interaction was described by ultrasoft pseudopotential with a cutoff energy of 400 eV. To relax the ions into their ground states, a conjugate-gradient algorithm was employed and the energy on each ion was less than 1.0 × 10^−5^ eV/atom. Total energy calculations were achieved when the residual force of less than 0.01 eV/Å. DFT simulations were performed based on a TiO_2_ (101) slab model system. The surface is constructed as slab based on the three-dimensional (3-D) periodic boundary conditions, and the vacuum layer about 12 Å was set in order to separate the models from their images in the perpendicular direction. For these calculations, a 5 × 5 × 1 *k*-Point mesh was used for the slab model [36].

## 3. Results and Discussion

### 3.1. Material Characterization

**Figure 1:**
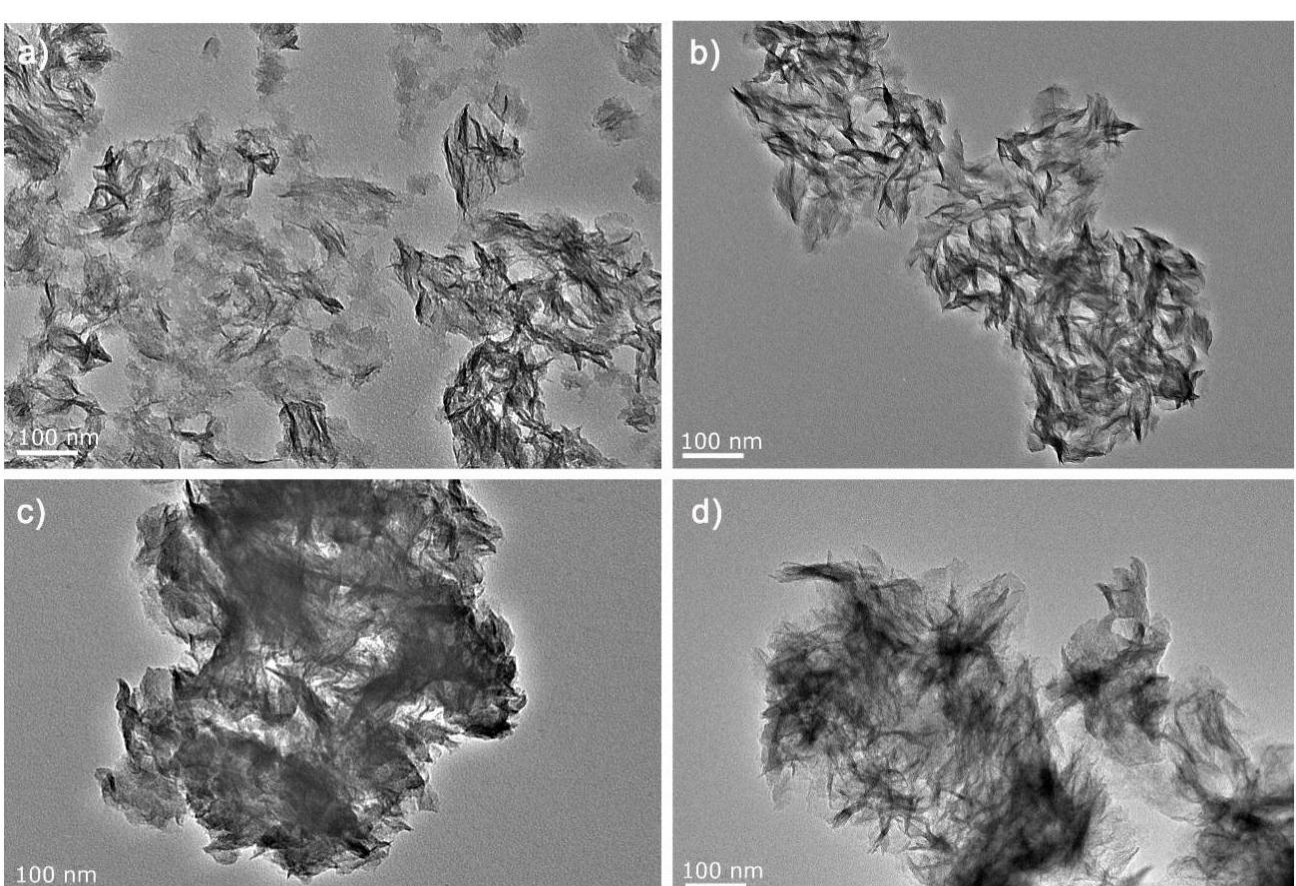
Representative transmission electron microscopy (TEM) images of (**a**) pure TiO_2_; (**b**) 10% F-TiO_2_; (**c**) 10% I-TiO_2_ and (**d**) 10% S-TiO_2_.

The surface morphology of the as-prepared pure and doped TiO_2_ nanosheets was analyzed using TEM. The pure TiO_2_ nanosheets consisted of wrinkled and nearly transparent nanosheets (Figure 1a). Plenty of edges were rolled up due to surface tension, indicating the ultrathin structure of TiO_2_ NSs [22,37]. The size of the TiO_2_ NSs is approximately 200 nm. According to Figure 1b–d, no apparent change could be observed on the TiO_2_ NSs after doping.

**Figure 2:**
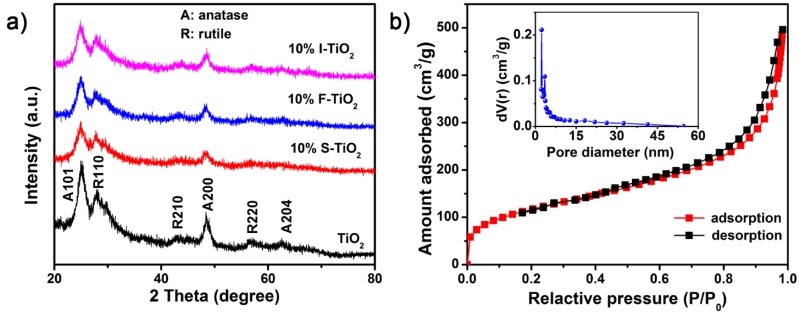
(**a**) X-ray diffraction (XRD) patterns of pure TiO_2_, 10% F-TiO_2_, 10% I-TiO_2_, 10% S-TiO_2_; (**b**) N_2_ adsorption-desorption isotherms of the pure TiO_2_ NSs, while the inset is the curve of pore size distribution.

As shown in the XRD patterns (Figure 2a), the diffraction peaks of all the samples are mainly composed of anatase-phase TiO_2_ (JCPDS No. 21-1272), and only traces of rutile-phase TiO_2_ (JCPDS No. 21-1276) can be observed [22,38]. Noted that the diffraction peaks are relatively broad and weak, since the graphene-like nanosheets lack long-range atomic order in the third dimension [39]. However, due to the small doping amount, the diffraction peaks of the doped TiO_2_ nanosheets were similar to that of the pure TiO_2_ except for a slight decrease in the peak intensities, which might be due to defects and surface modification on the TiO_2_ NSs [22,40]. No apparent impurity signal appeared after doping, indicating that the basic structure of TiO_2_ has not been changed. In addition, Nitrogen adsorption-desorption isotherms of the 2D TiO_2_ NSs are shown in Figure 2b, which revealed a high specific surface area of 422.86 m^2^·g^−1^ and an average pore size of ~7.3 nm. Therefore, the ultrathin 2D graphene-like nanosheets would provide more adsorption sites in comparison to micro-/nano-particle materials and could be a promising electrode modification material [41].

### 3.2. Experimental Parameters Optimization

**Figure 3:**
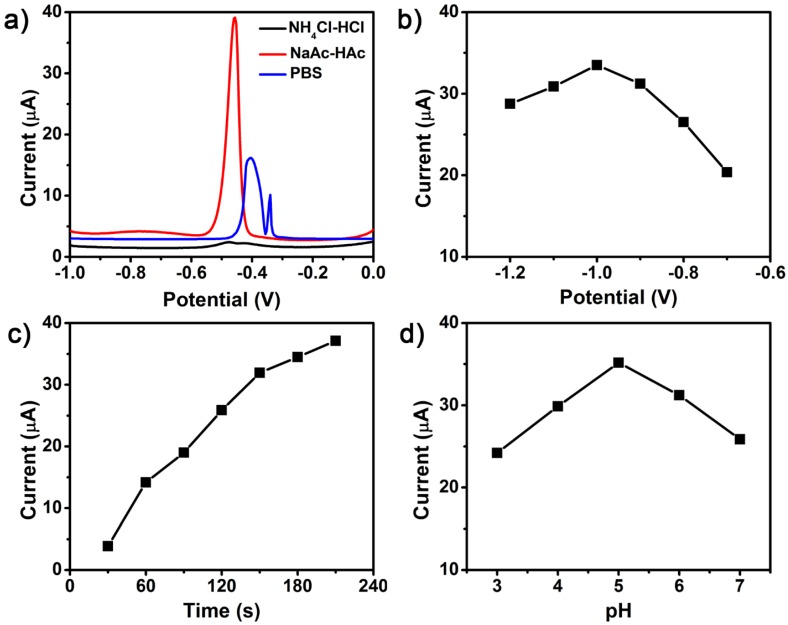
Influence of experimental parameters: (**a**) supporting electrolytes, (**b**) deposition potential, (**c**) deposition time and (**d**) pH values on Square wave anodic stripping voltammetry (SWASV) responses of the TiO_2_ modified glass carbon electrode (GCE) in 0.1 M NaAc-HAc containing 0.5 μM Pb(II).

In order to obtain high sensitivity for the detection of trace HMIs with TiO_2_ NSs modified GCEs, several key experimental parameters, such as supporting electrolytes, deposition potential, deposition time, and pH values, were first optimized and presented in Figure 3. Figure 3a shows the voltammetric behavior toward 0.5 μM Pb(II) using different supporting electrolytes (0.1 M, pH 5.0): PBS, NaAc-HAc, and NH_4_Cl-HCl. No apparent signal was exhibited in NH_4_Cl-HCl. A broad and heterogeneous peak appeared in PBS solution. However, the well-defined and strong stripping peak was observed in NaAc-HAc electrolyte. Hence, the 0.1 M NaAc-HAc electrolyte was applied in further experiments. Figure 3b shows the influence of deposition potential, which ranged from −0.7 to −1.2 V in NaAc-HAc solution. The peak currents increased as the deposition potential shifted from −0.7 to −1.0 V, and reached the maximum at −1.0 V. As the potential rose beyond −1.0 V, the response decreased, which was most likely caused by the interference of hydrogen evolution [13,42]. Therefore, −1.0 V was selected in the subsequent experiment. 

Figure 3c depicts the effect of deposition time (30, 60, 90, 120, 150, 180, and 210 s) on the stripping signal toward Pb(II). The peak currents increased with the deposition time, while the rising rate of the peak currents slowed down after the deposition time reached 150 s, which could possibly ascribe to the surface active sites saturation [43,44]. In terms of efficiency, 150 s was chosen as the deposition time in the subsequent experiments. The influence of the pH values on the electrochemical performance was studied in the pH ranging from 3.0 to 7.0, as displayed in Figure 3d. The peak currents for Pb(II) were gradually increased as pH value increased from 3.0 to 5.0, and the maximum current was observed at 5.0. As the pH value exceeded 5.0, the peak current declined dramatically, which possibly resulted from the hydrolysis of metal ions [43,44,45]. Thus, the supporting electrolyte at pH 5.0 was served as the final condition in the follow-up experiments.

### 3.3. Comparison of Electroanalytical Performance of Non-Metal Doped TiO_2_ NSs

**Figure 4:**
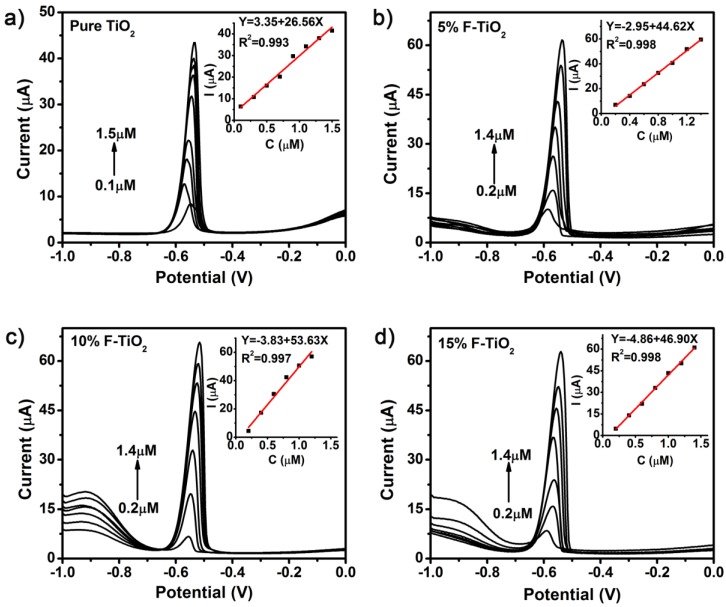
SWASV responses of (**a**) pure TiO_2_; (**b**) 5% F-TiO_2_; (**c**) 10% F-TiO_2_ and (**d**) 15% F-TiO_2_ modified electrodes toward Pb(II) at different concentrations in 0.1 M NaAc-HAc solution (pH 5.0). The insets show the corresponding calibration plots.

Figure 4 presents the SWASV profiles of the pure TiO_2_ and fluorine-doped TiO_2_ modified GCEs toward various concentrations of Pb(II) in NaAc-HAc solution. The results of the sulfur-doped and iodine-doped samples are shown in Figure S1. It can be seen that the well-defined peaks of Pb(II) was obtained with the peak currents of Pb(II) approximately at the potential of −0.55 V. As the concentration increases, the peaks shift to a more positive direction, which should be attributed to the overlap of the diffusion layers caused by the reoxidation from Pb(0) to Pb(II) [1]. The analytical curves for Pb(II) covered linear ranges varying from 0.2 to 1.4 μM, demonstrating the good linearity with the increasing Pb(II) concentrations. The calibration curves and the fitted correlation equations could be found in the corresponding illustrations. A further analysis of regression found that all of the residuals from the linear fitting in Figure 4 located in the range (−2, 2), and the coefficient of determination *R*^2^ were all larger than 0.99. These together demonstrate the accuracy and validity of linearity analysis. These results show that the non-metallic doping can enhance the electrochemical sensitivity toward Pb(II).

**Figure 5:**
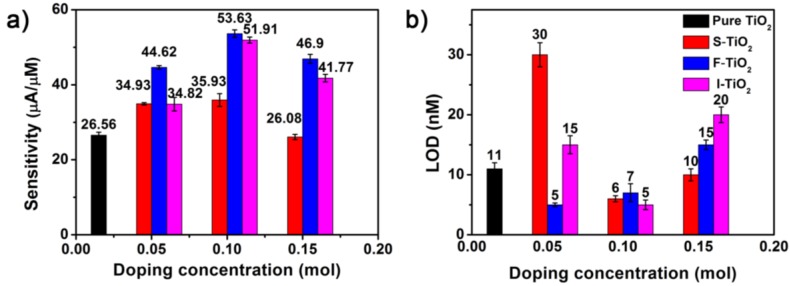
Comparison of (**a**) sensitivity and (**b**) limit of detection for SWASV detection of Pb(II) by pure TiO_2_, sulfur-, fluorine-, and iodine-doped TiO_2_ modified GCEs, respectively.

For clarity, Figure 5 summarizes the sensitivities and limits of detection (LODs, Signal/Noise = 3) of all the modified electrodes toward Pb(II) detection. In order to obtain the reliable results, we prepared three electrodes for each sample. The error bars representing the standard deviations of the three independent measurements have been included in Figure 5. The small variation indicates the good repeatability of the obtained results. The order of the sensitivities toward Pb(II) with different modified electrodes shown in Figure 5a is: fluorine-doped TiO_2_ > iodide-doped TiO_2_ > sulfur-doped TiO_2_ > pure TiO_2_. When compared with the pure TiO_2_ NSs, the sensitivity increases at least by 35% after S-doping, and the maximum enhancement can reach 102% with the electrode modified with 10% F-TiO_2_ NSs, indicating that element doping technology can greatly improve the sensing performance toward Pb(II). The LODs were also calculated and shown in Figure 5b, which were much lower than the provision by the World Health Organization for drinking water at 90 nM [46]. At this point, the fluorine-doped TiO_2_ is still the best among all the modified TiO_2_ nanosheets. When the doping concentration is 5%, the detection limit can decrease by 54.5% to 5 nM as compared to that of the bare TiO_2_ nanosheets. This performance was also compared with other reported electrodes (Table S1). Thus, the fluorine-doped TiO_2_ nanosheets present the superior improvement of sensitivity and limit of detection, which would be investigated in detail later to clarify the doping effects on the electrochemical analysis.

### 3.4. Study on the Reasons for Improved Electrochemical Properties of F-Doped TiO_2_

#### 3.4.1. Surface Chemical States Investigation

**Figure 6:**
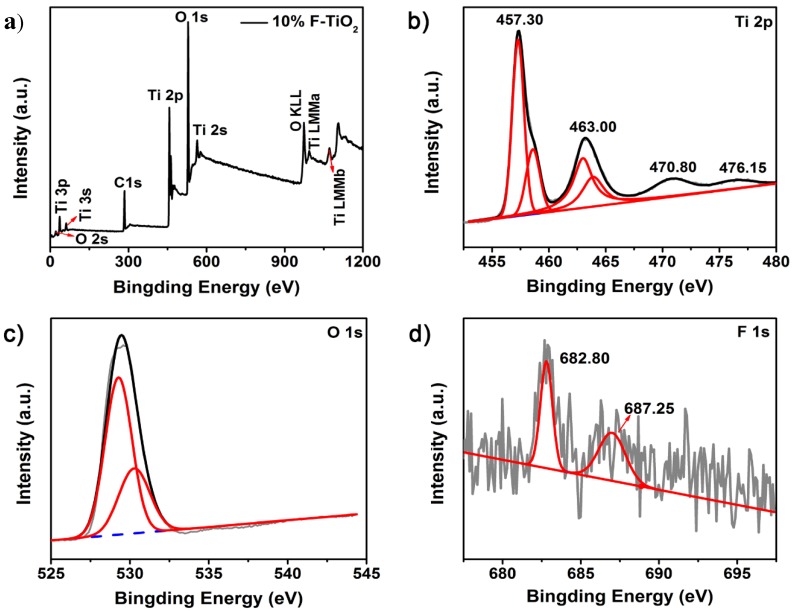
(**a**) Survey XPS spectrum of 10% F–TiO_2_ nanosheets; High-resolution XPS spectra of (**b**) Ti 2p; (**c**) O 1s and (**d**) F 1s regions for the 10% F–TiO_2_ nanosheets. The gray lines are original curves, and the red, black lines are the fitting curves.

To further explore the reasons for improving performance by element doping, the surface composition and chemical states of 10% F-TiO_2_ was investigated by XPS spectra. Figure 6a depicts the XPS survey scan for the 10% F-TiO_2_ nanosheets while Figure 6b–d present the high-resolution XPS spectra in Ti 2p, F 1s, and O 1s regions. Four XPS peaks in Figure 6b can be found in the range from 455 eV to 467 eV after fitting, where the peaks at 457.3 eV and 463.0 eV correspond to Ti^3+^ 2p_3/2_ and Ti^3+^ 2p_1/2_ states, and those at 458.6 eV and 464.0 eV to Ti^4+^ 2p_3/2_ and Ti^4+^ 2p_1/2_, respectively. This is consistent with the results of the previous reports [22,47,48]. It is worth noting that when compared with the corresponding bulk crystal, these peaks show a certain degree of deviation to lower binding energy. The lower binding energy for the ultrathin TiO_2_ nanosheets is the result of the redistribution of electron density around the titanium atoms due to the significant lattice distortion [22]. The lattice structural distortion of such atomically thin nanosheets have been reported and it is associated with extra chemical bond formation, fracturing, or bond elongation/contraction/deflection between the metal and oxygen atoms as compared to their three-dimensional bulk materials [22,39]. These results demonstrate the selected ultrathin 2D nanosheets structures with unique chemical states of the surface atoms, which might introduce some anticipated physiochemical properties that differ from traditional morphologies. 

The peaks at 529.28 and 530.36 eV in Figure 6c are assigned to the lattice oxygen atoms of TiO_2_ and Ti_2_O_3_, respectively [46]. Figure 6d presents the XPS peaks in F 1s regions. The peak at 682.8 eV indicates that the F^−^ physically adsorption on 10% F-TiO_2_ [49]. The other peak at 687.3 eV is assigned to the F atoms which substituted O atoms in TiO_2_ crystal lattice, which is due to the similar ion radii between F^−^ and O^2−^ so that the nucleophilic substitution reaction of F^−^ probably occurred during the hydrolysis of titanium alkoxide [49,50]. Therefore, F element has been successfully incorporated into the TiO_2_ crystal lattice.

#### 3.4.2. Further Electrochemical Characterization

**Figure 7:**
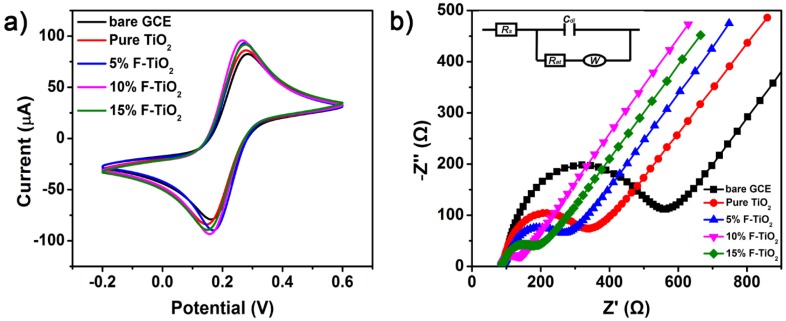
(**a**) CV and (**b**) EIS analysis of the pure TiO_2_, 5% F–TiO_2_, 10% F–TiO_2_, and 15% F–TiO_2_ modified GCEs in the solution of 5 mM Fe(CN)_6_^3−/4−^ containing 0.1 M KCl. The corresponding results of the bare GCE are also included for reference. The inset in Figure 7b is the equivalent circuit.

CV and EIS analysis have been further carried out to investigate the electrochemical features of various F–TiO_2_ modified electrodes. Figure 7a depicts the CV results of the bare, pure TiO_2_, 5% F–TiO_2_, 10% F–TiO_2_ and 15% F–TiO_2_ modified GCEs. For each electrode, we swept the 5 ring CV, where the CV scan became stable from the second lap, and the last lap was selected for comparison. The electrodes modified with TiO_2_ nanosheets show relatively larger current signals in comparison to the bare GCE. Among them, the 10% F–TiO_2_ modified electrode exhibits the highest peak current (*i_p_*) and the smallest peak potential separation (Δ*E_p_*). These further confirm that the modification of 10% F–TiO_2_ nanosheets on the electrode promotes the electron transfer between the electrode and the redox species and has a good current response performance. The interface features of the modified electrodes were further investigated using EIS. Figure 7b shows the Nyquist figures for the different modified electrodes, and the inset depicts the equivalent circuit, where *R_s_*, *C_dl_*, *W*, *R_et_* correspond to the solution resistance, the double layer capacitance, the Warburg impedance, and the electron-transfer resistance, respectively. It is well known that a typical Nyquist plot includes a semicircle portion and a linear portion. The semicircle portion at higher frequencies range corresponds to the electron transfer resistance (*R_et_*), and the linear part at lower frequencies range corresponds to the diffusion process [41]. It is found that the bare GCE has a relative large *R_et_* value of about 580 Ω. After modification, the *R_et_* values distinctly decrease, indicating that fluorine doping can accelerate the electron transfer on the electrode. Among all of the modified GCEs, 10% F–TiO_2_ nanosheets modified electrode shows the lowest *R_et_* value of 60 Ω, indicating that the fluorine doping could regulate the physiochemical properties of TiO_2_ nanosheets, which might accelerate the carrier transfer process between the electrolyte and the electrode.

**Figure 8:**
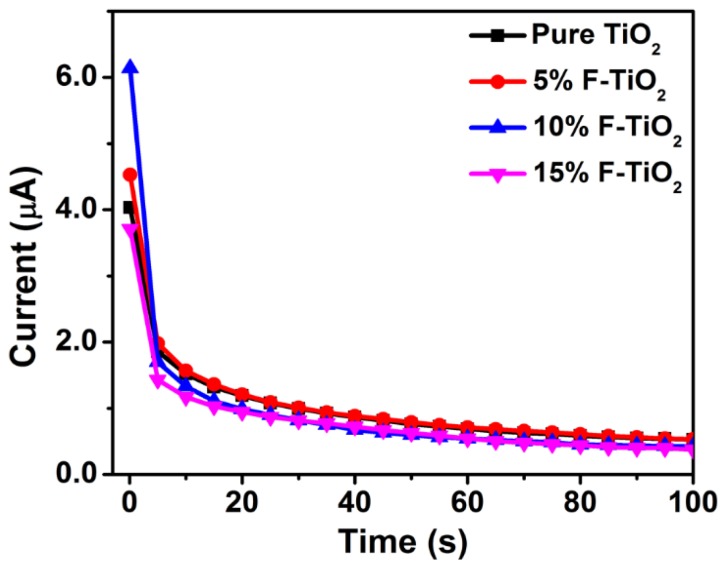
Dependence of stripping current of 1.0 μM Pb(II) on different desorption time for various modified GCEs.

The desorption capacity of the modified materials toward lead ions have also a profound effect on the electrochemical analysis. The corresponding desorption currents of the pure TiO_2_ and fluorine-doped TiO_2_ toward Pb(II) was tested at the potential of 0.3 V, and the results were exhibited in Figure 8. As the desorption time increases, the desorption current gradually decreases, indicating that the amount of Pb(II) gradually decreases on the electrode surface. Moreover, in the first 5 s the stripping current of the 10% F–TiO_2_ modified electrode is higher than those of the other electrodes, and it dramatically decrease by 83.94% to a minimum value. This is consistent with the discussion above, where the 10% F–TiO_2_ modified electrode presents an excellent stripping response toward Pb(II). In other words, Pb(II) on the 10% F–TiO_2_ modified electrode is easier to diffuse onto the electrode surface for reduction and stripping reaction than that on the pure TiO_2_, 5% F–TiO_2_ and 15% F–TiO_2_ modified electrodes. Therefore, the GCE modified with 10% F–TiO_2_ nanosheets can show higher sensitivity toward Pb(II). 

#### 3.4.3. Theoretical Calculations

**Figure 9:**
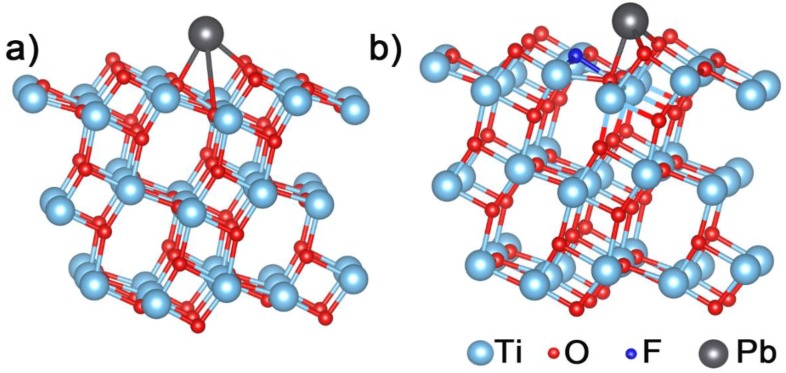
Side views of the optimized adsorption configurations of Pb(II) on (**a**) TiO_2_ and (**b**) F–TiO_2_ nanosheets exposed (101) facets.

To further verify the adsorption interaction of lead ion with TiO_2_ before and after doping, the DFT calculation was employed to simulate the adsorption behaviors of Pb(II) on TiO_2_ and 10% F–TiO_2_ surfaces in atomic level system. From the XRD results in Figure 2a, the content of anatase (101) is the highest. It is known that the average surface energies of anatase TiO_2_ follow the order: 0.90 J/m^2^ for {001} > 0.53 J/m^2^ for {100} > 0.44 J/m^2^ for {101} [51]. Based on surface energy and XRD results, the anatase type of titanium dioxide exposing (101) surface was adopted in our simulation. The slab model is consisted of 1 × 2 supercell, containing a total of 72 atoms (Ti atoms 24, O atoms 48). After optimizing geometry configurations, the most favorable adsorption sites on the TiO_2_ (101) and F–TiO_2_ (101) surfaces are depicted in Figure 9. For the adsorption of Pb(II) on the TiO_2_ (101) surface in Figure 9a, the Pb–O bond lengths are predicted to be about 2.28, 2.34, and 3.42 Å (summarized in Table 1). For Pb(II) on the F–TiO_2_ (101) surface in Figure 9b, the bond lengths of Pb–O are 2.22, 2.23, and 2.40 Å. The Pb–O bond lengths formed in the Pb/F–TiO_2_ (101) system are shorter than those in the Pb/TiO_2_ (101) system, which reflects stronger adsorbing interaction between Pb(II) and the F–TiO_2_ NSs. In addition, the equation, *E_ads_* = *E_Pb/slab_* − (*E_Pb_* + *E_slab_*), was employed to further obtain the adsorption energy. Here, *E_ads_* represents the Pb adsorption energy on the material surface, *E_Pb/slab_* is the total energy of interaction between a Pb atom and the slab, *E_Pb_* stands for the total energy of single Pb atom, and *E_slab_* is the energy of bare slab. The more negative that the calculated *E_ads_* value is, the stronger interaction of Pb(II) with the material surfaces. Table 1 displays the calculated adsorption energies of Pb(II) on the (101) surfaces of TiO_2_ and F–TiO_2_ nanosheets. The adsorption energies of Pb(II) on (101) surface of TiO_2_ and F–TiO_2_ were calculated as −2.23 and −2.65 eV, respectively. This indicates the greater adsorption capacity of the F–TiO_2_ (101) surface toward Pb(II) than TiO_2_ (101), which was in good agreement with the discussion above. Our experimental observations are quite supported by these theoretical calculation results, and that the best ASV behavior of the F–TiO_2_ nanosheets to Pb(II) can be well understood.

#### 3.4.4. Stability and Interference Studies

**Figure 10:**
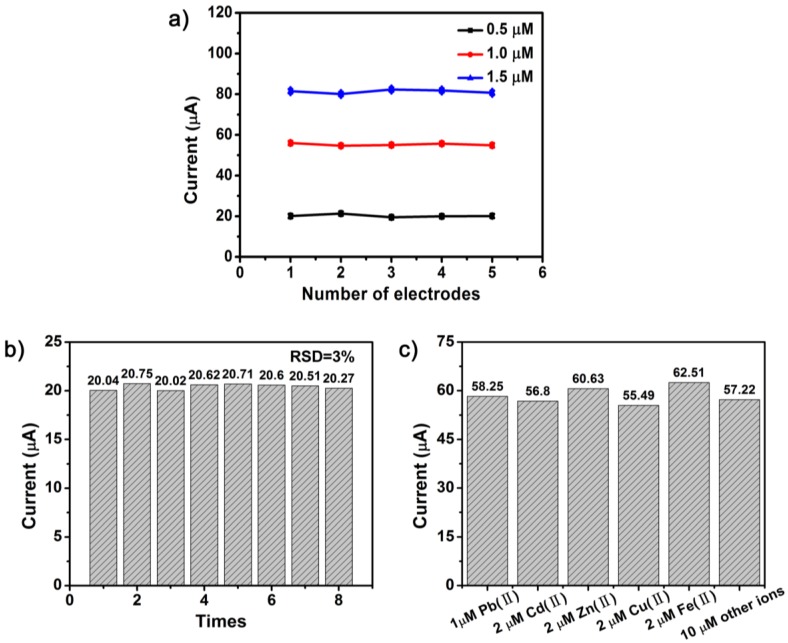
(**a**) Reproducibility valuation using three different 10% F–TiO_2_ modified GCEs in the solution of 0.5 μM, 1 μM, and 1.5 μM Pb(II); (**b**) Stability measurements to 0.5 μM Pb(II) by using the 10% F–TiO_2_ modified GCE; (**c**) Interferences of some ions on the stripping peak currents of 1 μM Pb(II) by using 10% F–TiO_2_ modified GCE. Other ions: Al^3+^, K^+^, Na^+^, Ca^2+^, Mg^2+^, NH^4+^, Cl^−^, NO^3−^, SO_4_^2−^, and PO_4_^3−^.

The reproducibility, long-term stability, and anti-interference performance of a good electrode material are also crucial for its practical application. Firstly, as shown in Figure 10a, the currents at the optimum conditions was determined using three 10% F–TiO_2_ modified electrodes prepared independently. The relative standard deviation was 0.59% for 0.5 μM Pb(II), 0.50% for 1 μM Pb(II), and 0.81% for 1.5 μM Pb(II). Secondly, the stability study was evaluated toward 0.5 μM Pb(II) with the 10% F–TiO_2_ modified electrode for eight times under the optimum experimental conditions. The peak current was recorded as shown in Figure 10b. The relative standard deviation (RSD) can be calculated as 3%. These results show that the 10% F–TiO_2_ modified electrode has excellent reproducibility and stability.

The interference study was tested in 1 μM Pb(II) standard solution with a certain amount of interference ions under the optimum condition. The results in Figure 10c show that double concentration of Cd^2+^ or Cu^2+^ in the mixed solution results in a slight decrease (less than 10%) of current responses toward Pb(II), which may be ascribed to the fierce competition for electrode surface active sites among these electrochemical deposited metals as well as the tendency to form the intermetallic compound between interfering ions and target metals [52]. Double concentration of Zn^2+^ or Fe^3+^ in the mixed solution results in a slight increase of current response of Pb(II). However, the addition of other interfering ions, like Al^3+^, Mg^2+^, Na^+^, NH^4+^, K^+^, Ca^2+^, Cl^−^, PO_4_^3−^, SO_4_^2−^, and NO^3−^ (10 μM, respectively) do not cause any significant interference for the detection of Pb(II). Overall, the F–TiO_2_ modified GCE reveals a high selectivity toward Pb(II), indicating that the F–TiO_2_ modified GCE is suitable for practical applications.

## 4. Conclusions

In the present investigation, element doping has been demonstrated to be a facile method to adjust the electrochemical properties of ultrathin TiO_2_ nanosheets, which can effectively regulate the electroanalytical performance toward heavy metal-ions detection. The sensitivity and LODs are compared by the introduction of three non-metallic elements (I, F, and S) into TiO_2_ nanosheets for electrochemical detection toward Pb(II). Fluorine-doped TiO_2_ nanosheets show the highest electrochemical sensitivity and the lower detection limit toward Pb(II) when the doping concentration is 10%. The sensitivity of the electrochemical detection toward Pb(II) could reach 53.63 μA/μM in the range of 0.2–1.4 μM (correlation coefficient, *R*^2^ = 0.997), and the obtained LOD is 7 nM, which is much lower than the guideline value in drinking water given by the World Health Organization (WHO). When compared with the pure TiO_2_ nanosheets, the sensitivity has been increased by 102%. Combining the theoretical calculations and systematic electrochemical experiments, the enhanced electrochemical performance stemming from element doping has been clarified for the ultrathin 2D nanosheets structure. Firstly, F doping greatly increases the adsorption energy toward Pb(II), which is beneficial for the increase of loading capacity toward heavy metal ions. Secondly, F doping facilitates the electron transfer to the electrode. Thirdly, F doping shows a better desorption capacity toward Pb(II), which is favorable for the Pb(II) diffuse onto the electrode. Therefore, the doping technology of two-dimensional TiO_2_ nanosheets is promising to open new opportunities to extend the materials in detection of heavy metal ions in the environment. The results obtained suggest that the fluorine-doped TiO_2_ nanosheets are promising nanomaterials, which possess excellent performance in electrochemically detecting heavy metal ions.

## Figures and Tables

**Table 1:** Adsorption energy and bond length of Pb(II) adsorbed on TiO_2_ and F–TiO_2_.

Cyrstal Surface	Bond Length (Å)	E_ad_ (eV)
TiO_2_-101	Pb–O 2.28 2.34 3.42	−2.2361
TiO_2_(F)-101	Pb–O 2.22 2.23 2.40	−2.6517

## Supplementary Materials

The following are available online at http://www.mdpi.com/2079-4991/7/10/327/s1, Figure S1: SWASV responses of S-TiO_2_, I-TiO_2_ samples, Table S1: Comparison of electrochemical performance of nanomaterials modified electrodes.
