# Supplementary material for: Regulation of the Electroanalytical Performance of Ultrathin Titanium Dioxide Nanosheets toward Lead Ions by Non-Metal Doping
Source: Nanomaterials (Basel). 2017 Oct 14;7(10):327. doi: 10.3390/nano7100327 (PMC5666492; doi:10.3390/nano7100327)
Supplement: Supplementary file 1 [file nanomaterials-07-00327-s001.docx]

Supporting Information

#### Regulation of the electroanalytical performance of ultrathin titanium dioxide nanosheets toward lead ions by nonmetal doping

Junping Zhang ^1,2,†^, Jianjun Liao ^1,3,†^, Fan Yang ^1,2^ , Ming Xu ^1,2^ and Shiwei Lin ^1,2,*^

^1^ State Key Laboratory of Marine Resource Utilization in South China Sea, Hainan University, Haikou 570228, P. R. China

^2^ College of Materials and Chemical Engineering, Hainan University, Haikou 570228, P. R. China

^3^ Institute of Tropical Agriculture and Forestry, Hainan University, Haikou 570228, China

† Contributed equally to this work

***** Correspondence: linsw@hainu.edu.cn (S. Lin)


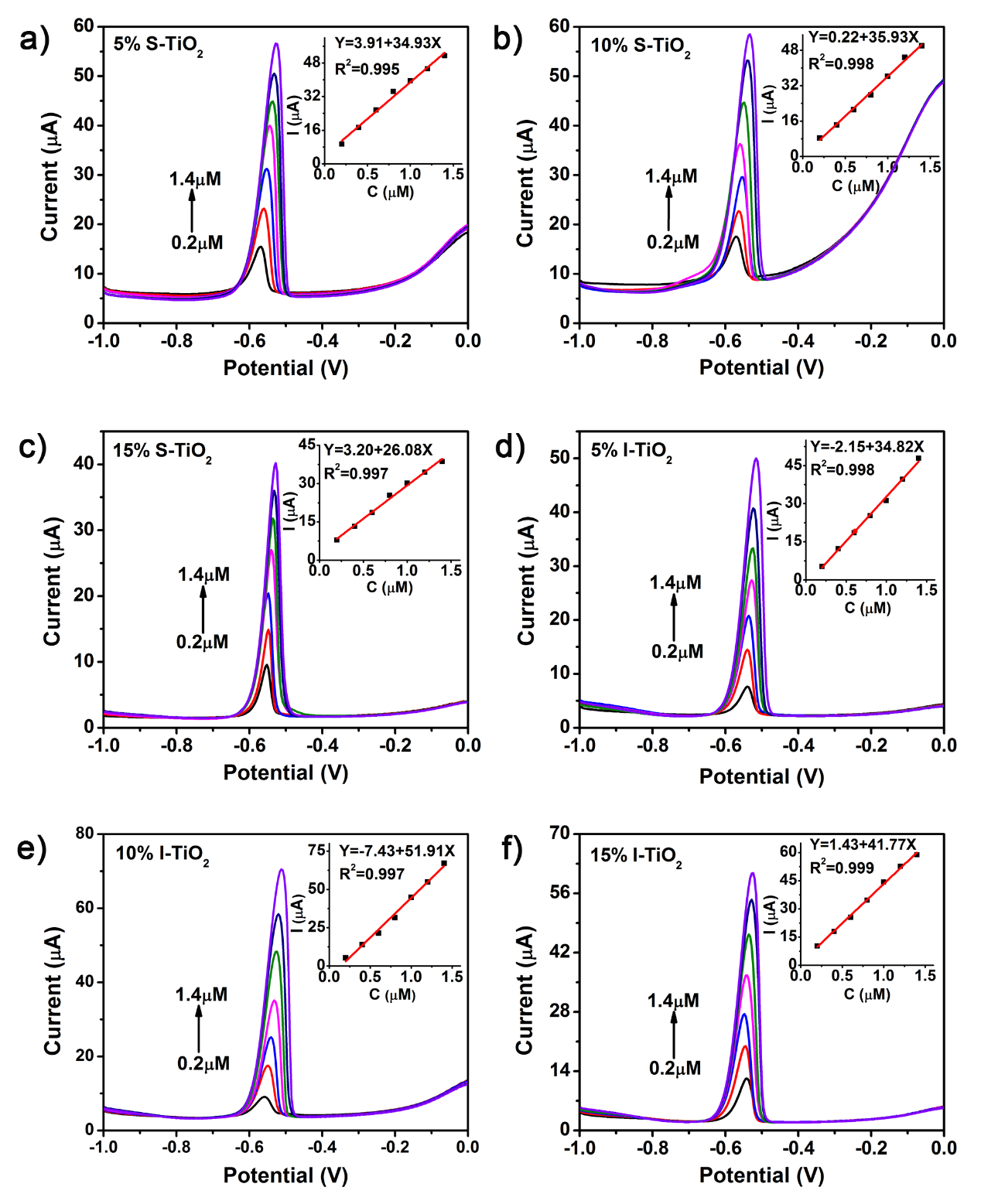


**Figure S1.** SWASV responses of (a) 5% S-TiO_2_, (b) 10% S-TiO_2_, (c)15% S-TiO_2_, (d) 5% I-TiO_2_, (e) 10% I-TiO_2_ and (f) 15% I-TiO_2_ modified electrode toward Pb(II) at different concentrations in 0.1 M NaAc-HAc solution (pH 5.0). The insets show the corresponding calibration plots.

**Table S1.** Comparison of electrochemical performance of nanomaterials modified electrodes for voltammetric determination of Pb(II).

| **Electrodes** | **Electrolyte** | **Deposition time(s)** | **linear range (μM)** | **LOD (nM)** | **Sensitivity (μA μM^-1^)** | **Ref** |
| --- | --- | --- | --- | --- | --- | --- |
| Fe_3_O_4_ nanocrystals  Nanosheets | 0.1 M HAc- NaAc (pH 5.0) | 120 | 0.1-1.6 | 12 | 5.52 | [1] |
| AuNPs/CNFs | 0.1M PBS | 150 | 0.1-1.0 | 100 | 19.08 | [2] |
| Cu _2_ O microcrystals | 0.1 M HAc- NaAc (pH 5.0) | 120 | 1.3-2.0 | 66 | 178μA cm^-2^uM^-1^ | [3] |
| PdNPs/PAC/GCE | 0.1 M HAc- NaAc (pH 5.0) | -- | 0.5-8.9 | 50 | 7.28 | [4] |
| [Ru(bpy)_3_]^2+^-GO/Au electrode | 0.1M citrate buffer at pH 5 | -- | 0.05-0.25 | 1.41 | 34.74 | [5] |
| Layered Co_3_O_4_ | 0.1 M HAc- NaAc (pH 5.0) | 180 | 0.05-0.275 | 52 | 28.26 | [6] |
| CNFs/GCE | 0.1 M HAc- NaAc (pH 4.5) | 300 | 0.2-1.0 | 0.9 | 57.8 | [7] |
| IL-CNT-GF | 0.1 M HAc- NaAc (pH 4.6) | 300 | 0.08-1 | 0.2 | 0.75 mA cm^-2^μM^-1^ | [8] |
| Lance-shaped SnO_2_/GCE | 0.1 M NaAc-HAc(pH 5.0) | 120 | 0.1-0.6 | 17 | 22.61 | [9] |
| F-doped TiO_2_ nanosheets | 0.1 M NaAc-HAc(pH 5.0) | 150 | 0.2-1.4 | 7 | 53.63 | This work |

LOD: limit of detection; GCE: glassy carbon electrode; AuNPs: Gold nanoparticles; CNFs: carbon nanofibers; PdNPs/PAC: Palladium nanoparticles on porous activated carbons; [Ru(bpy)_3_]^2+^-GO/Au: ruthenium(II)-textured graphene oxide nanocomposite; CNFs: carbon nanofiber; IL-CNT-GF: ionic liquid (IL)-carbon nanotube (CNT)-graphene film (GF); SWASV: square wave anodic stripping voltammetry; DPV：differential pulse voltammetry; ASV: anodic stripping voltammetry.

**References**

1. Yao, X. Z.; Guo, Z.; Yuan, Q. H.; Liu, Z. G.; Liu, J. H.; Huang, X. J. Exploiting differential electrochemical stripping behaviors of Fe_3_O_4_ nanocrystals toward heavy metal ions by crystal cutting. *ACS Appl. Mater. Interfaces* **2014**, *6*, 12203-12213.

2. Zhang, B.; Chen, J.; Zhu, H.; Yang, T.; Zou, M.; Zhang, M.; Du, M. Facile and green fabrication of size-controlled AuNPs/CNFs hybrids for the highly sensitive simultaneous detection of heavy metal ions. *Electrochim. Acta* **2016**, *196*, 422-430.

3. Liu, Z. G.; Sun, Y. F.; Chen, W. K.; Kong, Y.; Jin, Z.; Chen, X.; Zheng, X.; Liu, J. H.; Huang, X. J.; Yu, S. H. Facet-Dependent Stripping Behavior of Cu_2_O Microcrystals Toward Lead Ions: A Rational Design for the Determination of Lead Ions. *Small* **2015**, 2493-2498.

4. Veerakumar, P.; Veeramani, V.; Chen, S. M.; Madhu, R.; Liu, S. Bin Palladium Nanoparticle Incorporated Porous Activated Carbon: Electrochemical Detection of Toxic Metal Ions. *ACS Appl. Mater. Interfaces* **2016**, *8*, 1319-1326.

5. Gumpu, M. B.; Veerapandian, M.; Krishnan, U. M.; Rayappan, J. B. B. Simultaneous electrochemical detection of Cd(II), Pb(II), As(III) and Hg(II) ions using ruthenium(II)-textured graphene oxide nanocomposite. *Talanta* **2017,** 162, 574-582.

6. Liu, Z. G.; Chen, X.; Liu, J. H.; Huang, X. J. Well-arranged porous Co_3_O_4_ microsheets for electrochemistry of Pb(II) revealed by stripping voltammetry. *Electrochem. commun.***2013**, *30*, 59-62.

7. Zhao, D.; Wang, T.; Han, D.; Rusinek, C.; Steckl, A. J.; Heineman, W. R. Electrospun Carbon Nanofiber Modified Electrodes for Stripping Voltammetry. *Anal. Chem.***2015**, *87*, 9315-9321.

8. Dong, S.; Wang, Z.; Asif, M.; Wang, H.; Yu, Y.; Hu, Y.; Liu, H.; Xiao, F. Inkjet Printing Synthesis of Sandwiched Structured Ionic Liquid-Carbon Nanotube-Graphene Film: Toward Disposable Electrode for Sensitive Heavy Metal Detection in Environmental Water Samples. *Ind. Eng. Chem. Res.***2017**, *56*, 1696-1703.

9. Jin, Z.; Yang, M.; Chen, S.-H.; Liu, J.-H.; Li, Q.-X.; Huang, X.-J. Tin Oxide Crystals Exposed by Low-Energy {110} Facets for Enhanced Electrochemical Heavy Metal Ions Sensing: X-ray Absorption Fine Structure Experimental Combined with Density-Functional Theory Evidence. *Anal. Chem.***2017**, *89*, 2613-2621.
